# Comparative Genomics of DNA Recombination and Repair in Cyanobacteria: Biotechnological Implications

**DOI:** 10.3389/fmicb.2016.01809

**Published:** 2016-11-09

**Authors:** Corinne Cassier-Chauvat, Théo Veaudor, Franck Chauvat

**Affiliations:** Institute for Integrative Biology of the Cell, CEA, Centre Nationnal de la Recherche Scientifique (CNRS), Universite Paris-Sud, Université Paris-SaclayGif-sur-Yvette Cedex, France

**Keywords:** cyanobacteria, photoproduction, DNA recombination, DNA repair, genetic instability, insertion sequences, natural transformation, radiation resistance

## Abstract

Cyanobacteria are fascinating photosynthetic prokaryotes that are regarded as the ancestors of the plant chloroplast; the purveyors of oxygen and biomass for the food chain; and promising cell factories for an environmentally friendly production of chemicals. In colonizing most waters and soils of our planet, cyanobacteria are inevitably challenged by environmental stresses that generate DNA damages. Furthermore, many strains engineered for biotechnological purposes can use DNA recombination to stop synthesizing the biotechnological product. Hence, it is important to study DNA recombination and repair in cyanobacteria for both basic and applied research. This review reports what is known in a few widely studied model cyanobacteria and what can be inferred by mining the sequenced genomes of morphologically and physiologically diverse strains. We show that cyanobacteria possess many *E. coli*-like DNA recombination and repair genes, and possibly other genes not yet identified. *E. coli*-homolog genes are unevenly distributed in cyanobacteria, in agreement with their wide genome diversity. Many genes are extremely well conserved in cyanobacteria (*mutMS, radA, recA, recFO, recG, recN, ruvABC, ssb*, and *uvrABCD*), even in small genomes, suggesting that they encode the core DNA repair process. In addition to these core genes, the marine *Prochlorococcus* and *Synechococcus* strains harbor *recBCD* (DNA recombination), *umuCD* (mutational DNA replication), as well as the key SOS genes *lexA* (regulation of the SOS system) and *sulA* (postponing of cell division until completion of DNA reparation). Hence, these strains could possess an *E. coli*-type SOS system. In contrast, several cyanobacteria endowed with larger genomes lack typical SOS genes. For examples, the two studied *Gloeobacter* strains lack *alkB, lexA*, and *sulA*; and *Synechococcus* PCC7942 has neither *lexA* nor *recCD*. Furthermore, the *Synechocystis* PCC6803 *lexA* product does not regulate DNA repair genes. Collectively, these findings indicate that not all cyanobacteria have an *E. coli*-type SOS system. Also interestingly, several cyanobacteria possess multiple copies of *E. coli*-like DNA repair genes, such as *Acaryochloris marina* MBIC11017 (2 *alkB*, 3 *ogt*, 7 *recA*, 3 *recD*, 2 *ssb*, 3 *umuC*, 4 *umuD*, and 8 *xerC*), *Cyanothece* ATCC51142 (2 *lexA* and 4 *ruvC*), and *Nostoc* PCC7120 (2 *ssb* and 3 *xerC*).

## Introduction

Cyanobacteria, the oldest and most diverse Gram-negative bacteria (Shih et al., 2013) are the only prokaryotes capable of oxygen-evolving photosynthesis (Hamilton et al., 2016). They are viewed as the ancestors of plant chloroplasts (Archibald, 2009), and as major producers of (i) the Earth's oxygenic atmosphere (Schopf, 2011) and (ii) the carbonates sedimentary deposits (Bosak et al., 2013; Benzerara et al., 2014).

Contemporary cyanobacteria produce a tremendous quantity of oxygen, and fix CO2 (Jansson and Northen, 2010), NO_3_ and N_2_ (Zehr, 2011) into an enormous biomass that supports a large part of the food chain. N_2_-fixing cyanobacteria can be used to fertilize soils (Singh et al., 2016), in place of industrial N-fertilizers whose production consumes large amounts of fossil fuels (Grizeau et al., 2015). In colonizing a wealth of wastewater ecosystems that contain high levels of nitrate and phosphate (Abed et al., 2014) and/or heavy metals, cyanobacteria could be used for wastewater treatment (Abed et al., 2014; Singh et al., 2016).

Cyanobacteria produce a wealth of natural products that can influence human health (antioxidants, vitamins, antibacterial, toxins (Williams, 2009; Dittmann et al., 2015; Kleigrewe et al., 2016; Narainsamy et al., 2016). Hence, *Arthrospira* has served as a human food since time immemorial (Gao, 1998).

Cyanobacteria are also regarded as promising microbial factories for the production of chemicals from nature's most plentiful resources: solar light, water, CO_2_ (Lai and Lan, 2015; Savakis and Hellingwerf, 2015; Zhou et al., 2016). To reach this objective, it is necessary to (i) introduce and express in cyanobacteria the (heterologous) chemicals-producing genes they lack; (ii) redirect the photosynthetically-fixed carbon toward the production of the intended chemicals; (iii) increase the tolerance of the engineered cyanobacteria to the intended products and (iv) maintain, or increase, the genomic stability of the producer strains. These biotechnological works are mainly performed with the unicellular models *Synechocystis* sp. strain PCC6803, *Synechococcus* sp. strain PCC7942 (formerly *Anacystis nidulans* R2) and *Synechococcus* sp. strain PCC7002 (formerly *Agmenellum quadruplicatum* PR6) that possess a small sequenced and manipulable genome (http://genome.microbedb.jp/cyanobase/). These cyanobacteria can take up and incorporate extracellular DNA into their chromosome to create insertion, deletion, or replacement mutations (Orkwiszewski and Kaney, 1974; Stevens and Porter, 1980; Grigorieva and Shestakov, 1982). They can also be manipulated with replicative shuttle vectors derived from (i) their endogenous plasmids (Kuhlemeier et al., 1981; Buzby et al., 1983; Chauvat et al., 1986), or (ii) the non-cyanobacterial plasmid RSF1010 (Mermet-Bouvier et al., 1993). Interestingly, this promiscuous plasmid replicates also in *Thermosynechococcus elongatus* (Mühlenhoff and Chauvat, 1996), *Prochlorococcus marinus* sp. strain MIT9313 (Tolonen et al., 2006), *Leptolyngbya* sp. strain BL0902 and *Nostoc punctiforme* sp. strain ATCC29133 (also registered as PCC73102) (Huang et al., 2010; Taton et al., 2014). Such RSF1010-derived plasmids proved useful tools for *in vivo* studies of (i) gene expression (Marraccini et al., 1993; Mermet-Bouvier and Chauvat, 1994; Mazouni et al., 1998; Figge et al., 2000; Mazouni et al., 2003; Huang et al., 2010; Dutheil et al., 2012); (ii) cell division (Mazouni et al., 2004; Marbouty et al., 2009), DNA repair (Domain et al., 2004); (iii) hydrogen production (Dutheil et al., 2012; Sakr et al., 2013; Ortega-Ramos et al., 2014); (iv) insertion sequence (Cassier-Chauvat et al., 1997); and (v) redox metabolism and responses to heavy metals (Poncelet et al., 1998; Marteyn et al., 2009, 2013).

Because of their photoautotrophic lifestyle, cyanobacteria are strongly challenged by DNA damages generated by solar UV rays and photosynthesis (for review see Cassier-Chauvat and Chauvat, 2015), likely explaining their resistance to radiations. Furthermore, many cyanobacteria engineered for biotechnological purposes appeared to be genetically unstable in using DNA recombination to inactivate/eliminate the newly introduced genes of industrial interest. Hence, a better understanding of DNA recombination and repair in cyanobacteria could help increasing their robustness and the genetic stability of the engineered strains. This would represent an important contribution toward the development of an economically viable photo-biotechnology. In this perspective, we used a comparative genomic approach (Table 1 and Supplemental Table 1), to show that cyanobacteria possess a large number of genes homolog to *Escherichia coli* DNA recombination and repair genes, including the key SOS players *lexA* and *sulA*. The presence/absence of these genes and information concerning their function and/or regulation indicate that some cyanobacteria may possess an *E. coli*-like SOS-type DNA repair system. These findings do not exclude the possible existence in cyanobacteria of other DNA repair genes, not yet identified.

**Table 1:** **Reference of the genes from ***Synechocystis*** PCC6803 (sll or slr), ***E.coli*** (eco) or ***B.subtilis*** (BSU) in the MBGD data base (http://mbgd.genome.ad.jp/) used for searching their homologs in the studied cyanobacteria**.

**Name**	**Protein function**	**Gene id**
*uvrA*	UvrA, excinuclease ABC subunit A	slr1844
*uvrB*	UvrB, excinuclease ABC subunit B	sll0459
*uvrC*	UvrC, excinuclease ABC subunit C	sll0865
*uvrD*	UvrD, excinuclease ABC subunit C/helicaseII	sll1143
*recA*	RecA, recombinase A	sll0569
*recBec*	RecB exonuclease V (RecBCD complex), beta subunit	eco:B2820
*recBcy*	Contains hhH domain and of nuclease of recB family	sll1686
*recC*	recC exonuclease V (RecBCD complex), gamma chain	eco:B2822
*recD*	recD exodeoxyribonuclease V, subunit alpha/ TraA family helicase	eco:B2819
*recF*	Recombination protein F RecF	sll1277
*recG*	ATP-dependent DNA helicase RecG	slr0020
*recJec*	recJ ssDNA exonuclease, 5' –> 3'-specific	eco:B2892
*recJcya*	single-stranded-DNA-specific exonuclease RecJ	sll1354
*recN*	DNA repair protein RecN	sll1520
*recQec*	ATP-dependent DNA helicase RecQ	eco:B3822
*recQcy*	ATP-dependent DNA helicase RecQ	slr1536
*recR*	Recombination protein F RecF	slr1426
*recO*	DNA gap repair protein	sll/eco:B2565
		
*ruvA*	Holliday junction DNA helicase RuvA	sll0876
*ruvB*	Holliday junction DNA helicase RuvB	sll0613
*ruvC*	Holliday juction resolvase RuvC	sll0896
		
*mutH*	mutH methyl-directed mismatch repair protein	eco:B2831
*mutL*	mutL DNA mismatch repair protein	slr1199
*mutM*	Formamidopyrimidine-DNA glycosylase	slr1689
*mutS1*	DNA mismatch repair protein MutS	sll1165
*mutS2*	recombination and DNA strand exchange inhibitor protein	sll1772
*mutT*	DNA mismatch repair protein Mutator Mut_like protein	slr1134
*mutY1*	A/G specific adenin glycosylase yfhQ	eco:B2961
		
*umuC*	umuC translesion error-prone DNA polymerase V subunit;	eco:B1184
*umuD*	SOS response UmuD protein	sll5123
		
*lexA*	lexA SOS function regulatory protein	sll1626
		
*ssb*	ssb single-stranded DNA-binding protein	slr0925
*dinB*	DNA polymerase IV	eco:B0231
		
*comA*	competence protein comEA, comA	slr0197
*comE*	competence protein comEC, comEA comE	sll1929
*comFA*	Competence protein ComF operon protein1	BSU35470
*comFB*	Competence protein ComFB protein2	BSU35760
*comFC*	Competence protein ComFC protein 3	BSU35450
*phR*	phr deoxyribopyrimidine photolyase	slr0854
*alkB*	alkB oxidative demethylase of N1 or N3 methylcytosine DNA lesions	eco:B2212
		
*xerC*	integrase recombinase	slr0733
*ogt/ada*	O6 methylguanine transferase/ fused DNA binding transcritional regulator	eco:B1335 and BSU13540
		
*sulA*	sulA cell division inhibitor	slr1223
*radA*	sms DNA repair protein RadA	slr0448

## Results and discussion

### Genomic diversity of cyanobacteria

In colonizing most waters (fresh, brackish and marine) and soils, where they face various challenges (Cassier-Chauvat and Chauvat, 2015), cyanobacteria have developed as widely diverse organisms (Narainsamy et al., 2013). Their genomes differ in size (from 1.44 to 12.07 Mb), ploidy (from two to more than 20 chromosome copies per cell) or GC content (30–60%), probably as a result from gains and losses of genes transferred by plasmids, insertion sequences (Alam et al., 1991; Cassier-Chauvat et al., 1997) and/or cyanophages (Hess, 2011; Shih et al., 2013). Most cyanobacteria possess a single circular chromosome, ranging from 1.44 Mb in size (the marine symbiotic strain UCYN-A) to 12.07 Mb (*Scytonema hofmanni* PCC7110) (Dagan et al., 2013). The well-studied strain *Synechocystis* PCC6803 has a 3.57 Mb chromosome, with a 48% GC content (http://genome.microbedb.jp/cyanobase/) and a copy number of 10–50 (Labarre et al., 1989; Griese et al., 2011). For the other models the values are 2.69 Mb, 55% and 2–5 for *Synechococcus* PCC7942 (Mann and Carr, 1974; Griese et al., 2011; Watanabe et al., 2015); and 3.00 Mb, 50%, and likely 2–5 for *Synechococcus* PCC7002 (Griese et al., 2011; Watanabe et al., 2015). *Synechocystis* PCC6803 also has seven plasmids, ranging from 2.3 Kb (Chauvat et al., 1986) to 119vKb (http://genome.microbedb.jp/cyanobase/); *Synechococcus* PCC7942 has one plasmid (46 Kb); and *Synechococcus* PCC7002 has seven plasmids (4.8–186 Kb). Interestingly, *Cyanothece* ATCC51142 possesses two chromosomes (one circular, 4.39 Mb; and one linear, 0.4 Mb) and four plasmids (10–39 Kb), whereas the marine strains *Prochlorococcus* and *Synechococcus* have a small chromosome (1.6–2.7 Mb), and no plasmids (Scanlan et al., 2009).

As a consequence of their genomic diversity, cyanobacteria produce a wealth of metabolites (Dittmann et al., 2015; Kleigrewe et al., 2016), display different cell morphologies (Cassier-Chauvat and Chauvat, 2014) and can differentiate cells, akinetes and/or heterocysts, respectively dedicated to cell survival in adverse conditions (Chauvat et al., 1982) or the fixation of atmospheric nitrogen (Flores and Herrero, 2010).

### Cyanobacteria can be resistant to radiations

Because of their photoautotrophic lifestyle, cyanobacteria are strongly challenged by solar UV rays and reactive oxygen species generated by photosynthesis (Cassier-Chauvat and Chauvat, 2015). Consequently, *Synechocystis* PCC6803 and *Synechococcus* PCC7942 are found to be more resistant to UV than the (non-photosynthetic) bacterium *E. coli* where DNA repair is best known (Baharoglu and Mazel, 2014). *Synechocystis* PCC6803 is also more resistant to gamma rays than *Synechococcus* PCC7942 and *E. coli* in that order (the doses yielding 10% survival are 660, 230, and 130 Gy, respectively (Domain et al., 2004). Other cyanobacteria are even more radioresistant, almost as the champion bacterium *Deinococcus radiodurans* [100% survival at 5kGy (Moseley and Mattingly, 1971; Ito et al., 1983)]. These radiation-resistant cyanobacteria are *Chroococcidiopsis* [10% survival to 4–5 kGy of gamma rays (Billi et al., 2000)], three *Anabaena* strains [they can grow at 5 kGy (Singh et al., 2010)] and *Arthrospira* PCC8005 [it grows at 800 Gy (Badri et al., 2015)]. Thus, cyanobacteria might be used in the future for leaching (and/or sequestration) of radionuclides (Acharya and Apte, 2013).

### Cyanobacteria can be naturally competent for genetic transformation mediated by DNA recombinations

The naturally transformable cyanobacteria *Synechococcus* PCC7942, *Synechococcus* PCC7002, and *Synechocystis* PCC6803 can take up extracellular DNA and to recombine it into their own genome (Orkwiszewski and Kaney, 1974; Stevens and Porter, 1980; Grigorieva and Shestakov, 1982). This capability served to create a wealth of insertions, deletions or replacement mutations (Lai and Lan, 2015; Savakis and Hellingwerf, 2015; Zhou et al., 2016).

Natural transformation is best studied in *Bacillus subtilis* and *Helicobacter pylori* (Dorer et al., 2011). DNA transported into the cytosol by the Com proteins (com for competence) is integrated into the recipient genome by the RecA, RecG, and RuvABC recombination proteins.

The *com* genes (Table 1) are widely distributed in cyanobacteria (Supplemental Table 1). *Synechocystis* PCC6803, *Synechococcus* PCC7942, and *Synechococcus* PCC7002 harbor the *comAEF* genes (Supplemental Table 1). The *Synechocystis* PCC6803 genes *comA* and *comF* truly operate in transformation (Yoshihara et al., 2001), and *comF* is also involved in phototactic motility (Nakasugi et al., 2006). The role of *comE* could not be verified because the *comE*-depleted mutant dies rapidly (Yoshihara et al., 2001). By contrast, the *Prochlorococcus* cyanobacteria endowed with small genomes have no *comAEF* genes, excepted *P. marinus* MIT9303, and *P. marinus* MIT9313 that possess *comA, come*, and *ComF* (Supplemental Table 1). These strains also have the *recA, recG*, and *ruvABC* genes (Supplemental Table 1). We have verified in *Synechocystis* PCC6803 that *ruvB* operates in genetic transformation (Domain et al., 2004). These finding suggest that *P. marinus* MIT9313 may be transformable in appropriate conditions.

Recently, the CRISPR/Cas9 genome editing system, which enhances the recombination efficiency and accelerates the process for chromosome segregation, was used for efficient genome editing in cyanobacteria (Li et al., 2016; Wendt et al., 2016).

### Cyanobacteria genetically engineered for biotechnological purposes can be genetically instable

Microbial organisms can genetically adapt themselves to their “laboratory” environment. This phenomenon explains the phenotypic differences observed between various sub-strains of the same organism cultivated in diverse laboratories. Hence, the four laboratory sub-strains of *Synechocystis* PCC6803 with different cell motility and/or ability to feed from glucose, harbor mutations, insertion or deletion, as compared to each others (Okamoto et al., 1999; Kanesaki et al., 2012; Trautmann et al., 2012).

Genetic instability can also be observed in strains genetically engineered for the synthesis of chemicals, where it can decrease the amplitude and/or durability of production. Genetic instability correlates with the toxicity of the products, and homologous recombination between repeated DNA motifs (Gellert and Nash, 1987; Holder et al., 2015), which are frequent in cyanobacteria (Elhai, 2015).

In the 61 articles reporting the genetic engineering of a model cyanobacterium for the synthesis of a biotechnological product, the level of production were analyzed only during short periods of times (usually not more than 30 days after the generation of the producer strains; Lai and Lan, 2015). Consequently, we know very little regarding genome (in)stability in engineered cyanobacteria growing under laboratory conditions. This genome (in)stability is an important issue in large industrial cultures that require many cell divisions of the engineered cyanobacteria. The longer the cultivation, the higher the probability of selecting spontaneous mutations decreasing the synthesis of the product to increase cell fitness.

A few studies reported the genetic instability of engineered cyanobacteria. We observed this phenomenon while attempting to use *Synechocystis* PCC6803 for the production of a uniformly ^14^C-labeled mouse urokinase (a serine protease). The urokinase producing plasmid, which replicated stably in the *recA*^−^ mutant of *E. coli*, invariably lost part of the urokinase gene upon propagation in *Synechocystis* PCC6803 (Chauvat et al., 1988). Another *Synechocystis* PCC6803 strain harboring *Pseudomonas aeruginosa* genes cloned its chromosome (at the *slr0168* neutral docking site) for lactic acid production, happened to rescue its growth by introducing a duplication (~160 bp) that generated premature stop codons into the *Pseudomonas* (NADPH/NADH) transhydrogenase gene (Angermayr et al., 2012).

Similarly, the *Synechococcus* PCC7942 strain harboring the *Pseudomonas syringae* gene (*efe*) encoding the ethylene-forming enzyme (Fukuda et al., 1992; Sakai et al., 1997), managed to introduce short nucleotide insertions in *efe* to stop ethylene production and recover a healthy growth (Takahama et al., 2003). Another recombinant *Synechococcus* PCC7942 strain could introduce a missense mutation in the *E. coli atoD* gene (acetoacetyl-CoA transferase) to decrease isopropanol production (Kusakabe et al., 2013).

In *Synechococcus* PCC7002, a recombinant strain managed to loose mannitol synthesis and recover healthy growth, in introducing a single-base deletion generating a stop codon in its *E. coli* mannitol-1-phosphate dehydrogenase *mtlD* gene (Jacobsen and Frigaard, 2014).

The *Synechocystis* PCC6803 and *Synechococcus* PCC7002 recombinant strains producing the *Zymomonas mobilis* pyruvate decarboxylase enzyme (PDC) for ethanol production, could introduce mutations, insertions, deletions or mobile genetic elements (insertion sequences) into the *pdc* gene to stop ethanol production (Schulze et al., 2015).

Insertion sequences (ISs) are approximately 1 kbp long DNA segments found in the genome of most living organisms, where they can interrupt genes (Bennett, 2004). Generally, an IS comprises an inverted repeat DNA sequence flanking one or two genes encoding the mobilization protein (transposase), which drives the excision and reinsertion of IS in genomes.

Many cyanobacterial chromosomes and/or plasmids harbor a few or numerous copies of ISs, as the widely distributed IS families IS4, IS5, IS630 and IS200-605, which are regarded as ancestral (Lin et al., 2011). Though several *P. marinus* strains harboring a small genome have no IS, the frequencies of IS do not systematically increase with the genome size. Indeed, IS represent 10% of the 5.8 Mb genome of *Microcystis aeruginosa* NIES843, 1.5% of the 3.95 Mb genome of *Synechocystis* PCC6803, and 1% of the 7.2 Mb genome of *Nostoc* (*Anabaena*) PCC7120 (Lin et al., 2011). Consistent with the findings that transposase genes can be induced by stresses (Hernández-Prieto et al., 2016), several studies employing a positive selection procedure showed that ISs can be truly mobile in cyanobacteria. First, a recombinant *Nostoc* (*Anabaena*) PCC7120 strain harboring a plasmid encoding the *B. subtilis* SacB enzyme (levan sucrase), which kills cells incubated in the presence of sucrose, generated sucrose resistant mutants resulting from the disruption of the *sacB* gene by a mobile IS895 element (Alam et al., 1991).

Similarly, an IS5 element of *Synechocystis* PCC6803 was shown to be mobile in rescuing the growth of a conditionally lethal mutant by disrupting the repressor gene that normally blocks the transcription of an essential ferredoxin-encoding gene (Cassier-Chauvat et al., 1997; Poncelet et al., 1998). Other recently transposed IS4 elements were identified through Southern blotting and DNA sequencing analysis of three *Synechocystis* PCC6803 sub-strains (Okamoto et al., 1999).

In addition, the presence of multiple copies of an IS in a genome can promote homologous recombination, leading to genome rearrangements (inversions or deletions; Gellert and Nash, 1987) that can modify cell fitness. Moreover, ISs can be transferred between genomes by horizontal gene transfer mechanisms. Thus, ISs are an important force in genome evolution (Bennett, 2004).

So far very few studies attempted to decrease or eliminate the negative influence of IS on biotechnological production. In *Corynebacterium glutamicum*, the deletion of two major IS elements generated a cell chassis with an increased ability to stably produce recombinant proteins (Choi et al., 2015). A similar strategy could be tested in the genetically manipulable cyanobacteria *Synechococcus* PCC7942 and *Synechococcus* PCC7002 because they possess only one and ten transposase genes, respectively (http://genome.microbedb.jp/cyanobase/). In contrast, an IS-deletion strategy is not an appealing for *Synechocystis* PCC6803 that possesses 128 transposase genes.

In *E. coli*, the stable propagation of recombinant DNA (usually cloned in plasmids) is achieved in strains where *recA*, the key DNA-recombination gene (Baharoglu and Mazel, 2014), has been inactivated to prevent unexpected DNA rearrangements. All cyanobacteria possess a *recA* gene (*Acaryochoris marima* MBIC11017 has 7 *recA* genes, Supplemental Table 1). The *recA* gene appeared to be indispensable to cell life in *Synechococcus* PCC7002 (Murphy et al., 1990), whereas it could be deleted from all chromosome copies in *Synechocystis* PCC6803 (Minda et al., 2005). The *Synechocystis* PCC6803 *recA* null mutant is bound to be of limited biotechnological interest because it is not only sensitive to UV-C, but also to standard fluence of white light required for cell growth. Furthermore, in being defective in DNA recombination a *recA*^−^ mutant is not appropriate for genetic manipulation of the cyanobacterial chromosome (cloning of heterologous genes encoding the synthesis of biotechnological products and/or deletion of endogenous genes limiting the intended production).

An interesting way to limit genetic instability of engineered bacteria is to clone the product-synthesizing genes under the control of regulatable expression signals to afford a user-controlled synthesis of the potentially harmful product. Using such regulatory signals, one can grow the engineered strain up to a large biomass, before triggering the synthesis of the intended product, which, otherwise, could have impaired the fitness and/or the genetic stability of the producer.

In cyanobacteria gene expression can be regulated by (i) light (*psbA2* promoter), (ii) the IPTG metabolite (*lac* promoter/repressor system), (iii) metals [cyanobacterial promoters *coaT, ziaA, etc* (Berla et al., 2013; Zhou et al., 2016)], or (iv) the growth temperature [lambda phage *p*R promoter controlled by the *c*I857 temperature-sensitive repressor (Ferino and Chauvat, 1989; Mermet-Bouvier and Chauvat, 1994)]. As put forward by other workers (Berla et al., 2013) an ideal system should combine the following properties.

- “It should be inactive in absence of inducer”;
- “It should produce a predictable response to a given concentration of a regulator”;
- “The inducer should have no harmful effect on the host organism”;
- “The inducer should be cheap and stable under the growth conditions of the host”;
- “The inducible system should act orthogonally to the host cell's transcriptional program (ideal transcriptional repressors should not bind to native promoters.)”

In our laboratory, we often used the temperature-controlled system that appeared to combine most of these advantageous properties (Dutheil et al., 2012; Marteyn et al., 2013; Ortega-Ramos et al., 2014) and references therein. This system tightly controls gene expression proportionally to growth temperatures i.e., absence of expression at temperature ≤30°C (the standard growth temperature of our favorite cyanobacterium *Synechocystis* PCC6803); intermediary expression at intermediate temperature 34–37°C; and strong expression at 39°C (where *Synechocystis* PCC6803 keep growing well). For instance, when this system was used to control the production of the heterologous enzymes chloramphenicol-acetyl-transferase and beta-galactosidase, which possess an easily quantified activity, the values were respectively ≤3 units (30°C); 700–1000 units (34–37°C) and 2000–4000 units (39°C) (Ferino and Chauvat, 1989; Mermet-Bouvier and Chauvat, 1994). Hence this system can be also used for basic research that requires the construction of conditionally-lethal mutants (Poncelet et al., 1998; Sakr et al., 2013).

### Distribution of direct DNA-damages reversal genes in cyanobacteria

From bacteria to higher eukaryotes, cells are continuously exposed to DNA damages generated by their own metabolism (Imlay, 2013) and/or exogenous sources (radiations, chemicals, etc). DNA lesions are repaired by conserved pathways that have been extensively studied in *E. coli* (Baharoglu and Mazel, 2014). The simplest system, the direct damage reversal pathway, removes only the base-modifying agent in one single step (Resende et al., 2011) catalyzed by the AlkB demethylase, the Ogt alkyltranferase, and the Phr (photorepairs of pyrimidine) photolyase.

Using a comparative genomic approach, we found that the 76 cyanobacterial genome sequences in the MBGD data base (http://mbgd.genome.ad.jp/) possess many genes orthologous to *E. coli* DNA recombination and repair genes. The *phr, alkB* and *ogt* orthologs (Table 1) are distributed unevenly in cyanobacteria (Supplemental Table 1). The *phr* gene is present in almost all cyanobacteria including some, but not all, *P. marinus* strains endowed with a small genome (1.6–2.7 Mb). In agreement with the light fluence they receive in their oceanic biotopes (Biller et al., 2015), the high-light-adapted strains *P. marinus* MIT9515 and *P. marinus* MED4 possess *phr*, whereas the low-light-adapted strains *P. marinus* MIT9303 and *P. marinus* MIT9313 lack *phr* (Supplemental Table 1), and are light sensitive (Biller et al., 2015). The *alkB* and *ogt* genes are less frequent than *phr*. All three genes *alkB, ogt*, and *phr* are simultaneously present in several (twelve) studied cyanobacteria, such as *Nostoc* (*Anabaena*) PCC7120 (filamentous), and *Cyanothece* PCC7425 (unicellular) where *ogt* is duplicated. The other (evolutionary distant) unicellular models *Synechocystis* PCC6803, *Synechococcus* PCC7942, and *Synechococcus* PCC7002 possess *phr* (Supplemental Table 1). *Synechocystis* PCC6803 has *alkB* but not *ogt, Synechococcus* PCC7942 has *ogt* (duplicated) but not *alkB*, and *Synechococcus* PCC7002 has neither *alkB* nor *ogt*. Interestingly, the symbiotic (marine) cyanobacterium UCYN-A has no *phr, alkB*, and *ogt*, in agreement with the fact that it possesses the smallest genome (1.44 Mb). The other symbiotic strain *Acaryochloris marina MBIC11017* endowed with a larger genome (8.36 Mb) has two *alkB*, three *ogt* (including one on a plasmid) but no *phr* (Supplemental Table 1).

### Distribution of nucleotide excision DNA repair genes in cyanobacteria

This pathway removes distortions of the double helix of DNA (pyrimidine dimers or DNA intra-strand cross-links), by excising a small group of bases (Baharoglu and Mazel, 2014). In *E. coli* the two-proteins complex UvrAB recognizes the DNA lesion; UvrC generates a double incision on both sides of the lesion and the UvrD helicase removes the single-strand DNA carrying the lesion. The missing DNA is re-synthesized by the DNA polymerase I (Pol I), and subsequently sealed by a ligase.

All tested cyanobacterial genomes possess the *uvrABCD* single-copy genes (Supplemental Table 1), where *uvrA* and *uvrB* are not organized in operon (Supplemental Figure 1), unlike what occurs in *E. coli*. In some cyanobacterial genomes *uvrA, uvrB, uvrC*, and/or *uvrD* are clustered with another DNA repair gene, such as *phr* or *recN* (gene clusters a and c in Supplemental Table 1 and Supplemental Figure 1). In the radiation-resistant cyanobacterium *Arthrospira* PCC8005, *uvrBCD* were found to be upregulated by gamma rays (no information is provided for *uvrA*) (Badri et al., 2015).

### Distribution of methyl-directed DNA mismatch repair genes in cyanobacteria

This pathway corrects the mispaired DNA bases generated by replication errors (Putnam, 2016). In *E. coli*, MutS recognizes mispaired DNA bases and coordinates with MutH and MutL (nucleases), MutM, MutT and MutY (DNA glycosylases) and UvrD (helicase) to direct excision of the newly synthesized DNA strand (not yet methylated at GATC sites by the Dam methylase) up to the mismatch. The resulting gap is filled up by a DNA polymerase (likely PolIII) and a ligase (Putnam, 2016).

All tested cyanobacteria have *mutM* (Supplemental Table 1), which was shown in *Synechococcus* PCC7942 to operate in resistance to high light (Mühlenhoff, 2000). All cyanobacteria possess *mutS*, which occurs in two copies, excepted in *Crinalium epipsammum* PCC 9333 (Supplemental Table 1). By contrast, *mutH* is absent in all cyanobacteria. The genetic diversity of cyanobacteria is well illustrated with the presence/absence of *mutL, mutt*, and *mutY* (Supplemental Table 1), which lies in front of *recR* in a few cyanobacterial genomes (Table 1 and Supplemental Figure 1). Several *P. marinus strains* lack *mutL, mutt*, and *mutY* (Supplemental Table 1). In *Arthrospira* PCC8005 (radiation-resistant) *mutST* were upregulated by gamma rays (Badri et al., 2015).

The model strains *Synechocystis* PCC6803, *Synechococcus* PCC7942, *Synechococcus* PCC7002 and *Nostoc* (*Anabaena*) PCC7120 possess *mutL, mutM, mutS* (duplicated), *mutT* (excepted *Synechococcus* PCC7002), *mutY* (excepted *Synechococcus* PCC6803 and *Nostoc* PCC7120) (Supplemental Table 1). Thus, *Synechococcus* PCC7942 is best suited to study all these genes through deletion/over-expression in the otherwise same genetic context.

### Distribution of recombinational DNA repair genes in cyanobacteria

This pathway repairs double-stranded breaks and cross-links. In *E. coli*, single-strand DNA nicks are enlarged by the RecQ helicase and RecJ exonuclease, into gaps that are recognized by the proteins RecFOR. The double-strand DNA breaks (DSB) are recognized by the RecBCD proteins that form an exonuclease/helicase complex. Subsequently, the RecFOR/RecBCD complexes (and RecN) load RecA to initiate homologous recombination and DNA repair. RecA mediates synapsis, forming a Holliday junction. Replication fills gaps. RecG, Ssb (single-stranded DNA binding protein) and RuvAB mediate branch migration (stimulated by RadA), and RuvC resolves the junctions (Baharoglu and Mazel, 2014).

DNA recombination also involves the XerC-XerD complex. It converts dimers of the chromosome into monomers to permit their segregation during cell division, and it contributes to the segregational stability of plasmids (Resende et al., 2011; Buljubašic et al., 2013).

In many bacteria, such as *H. pylori* and *B. subtilis* the AddA and AddB proteins replace RecB and RecC, respectively (Dorer et al., 2011; Wigley, 2013).

All cyanobacteria contain *recA*, which occurs as seven copies in the large genome (8.36 Mb) of *A. marina* MBIC11017. Four of these *recA* genes, possibly originating from gene duplication (Swingley et al., 2008), are located on four separate plasmids, while the other *recA* belong to the chromosome (Supplemental Table 1).

Like *recA, radA* and *recG* are present in all cyanobacteria, and *radA* is duplicated in *Cyanothece* PCC7425, *M. aeruginosa NIES-843* and UCYNA (Supplemental Table 1). It is the only duplicated gene in the very small UCYNA genome (1.44 Mb).

Many cyanobacteria have two copies of *recJ* and *recQ* genes. They are noted as *recJ*_*ec*_ or *recJ*_*cy*_, or *recQ*_*ec*_ or *recQ*_*cy*_ (ec for *E. coli*, cy for cyanobacteria), according to their high (*recJ*_*ec*_, *recQ*_*ec*_) or low (*recJ*_*cy*_, *recQ*_*cy*_) sequence similarity with their *E. coli* counterparts (Table 1 and Supplemental Table 1). This is true for *Synechococcus* PCC7002 and *Nostoc* PCC7120, where these duplicated genes can be studied and compared through deletion/over-expression. In *Arthrospira* PCC8005 (radiation-resistant), *recGJQ* were found to be upregulated by gamma rays (Badri et al., 2015). In contrast a few cyanobacteria has neither *recJ* nor *recQ*, as *P. marinus* MIT9515 (Supplemental Table 1). Also interestingly, the low-light-adapted *P*. marinus MIT9313 and *P. marinus* MIT9303 possess the *recQ* genes (and *ogt* and the competence genes *comE* and *comFC*), which are not present in other *Prochlorococcus* (Supplemental Table 1). In addition, both *P. marinus* MIT9313 and *P. marinus* MIT9303 lack the *phr* gene, which occurs in other *Prochlorococcus* (Supplemental Table 1), in agreement with their light-sensitivity (Biller et al., 2015). Collectively, these findings support the proposal that *P. marinus* MIT9303 and *P. marinus* MIT9313 belong to the same clade, which diverged early from the other *Prochloroccus* clades (Sun and Blanchard, 2014; Biller et al., 2015).

Almost all cyanobacteria have the single-copy genes *recF, recO* and *recR*, excepted *Cyanobacterium aponinum* PCC10605, *C. epipsammum* PCC9333 and *Cylindrospermum stagnale* PCC7417 which lack *recR* (Supplemental Table 1)

The *recBCD* genes are less conserved in cyanobacteria. For instance, the strain UCYN-A that possesses *recFOR* has no *recBCD* genes (Supplemental Table 1). Most *P. marinus* strains and several marine *Synechococcus* strains possess *recBCD*. Most of these strains possess two *recB* copies, noted *recB*_*ec*_ (good similarity with *E. coli recB*) or *recB*_*cy*_ (cy for cyanobacteria, low similarity with *E. coli recB*). In these strains, *recB*_*ec*_ belongs to the same genomic region than *recC* and *recD* (cluster f in Supplemental Table 1 and Supplemental Figure 1). In a few other cyanobacteria *recD* is duplicated (*Microcoleus* PCC7113) or triplicated (*A. marina* MBIC11017 and *N. punctiforme* PCC73102), irrespectively of the presence /absence or *recB*_*ec*_ and *recB*_*cy*_ (Supplemental Table 1). The well-studied model cyanobacteria lack *recB, recC*, or *recD*. Both *Synechocystis* PCC6803 and *Nostoc* (*Anabaena*) PCC7120 lack *recB*_*ec*_ and *recC*, while both *Synechococcus* strains PCC7942 and *Synechococcus* PCC7002 lack *recCD*.

The *recN* gene is present in all cyanobacteria to the noticeable exception of *Chamaesiphon minutus* PCC6605. Interestingly the RecN protein was absent in mature heterocysts of *Anabaena* PCC7120, the differentiated nitrogen-fixing cells that have lost the ability to divide (Hu et al., 2015).

In some cyanobacteria a few *rec* genes are clustered together (*recBCD* see cluster f in Supplemental Table 1 and Supplemental Figure 1), or with other DNA repair genes, including *uvrA* (cluster a) or *mutY* (cluster n; Supplemental Table 1 and Supplemental Figure 1).

All cyanobacteria have a *ssb* gene, which is repeated in a few strains. For instance, *ssb* is duplicated in *Nostoc* (*Anabaena*) PCC7120 and *A. marina* MBIC11017), while it is triplicated in *Chroococcidiopsis thermalis* PCC7203 and quadruplicated in *Cyanothece* PCC7822 (Supplemental Table 1). In these cyanobacteria (excepted *Nostoc* (*Anabaena*) PCC7120) one *ssb* copy is propagated on a plasmid. One of the two *Nostoc* PCC7120 *ssb* genes, (*alr0088*, but not *alr7579*) was shown to be involved in the tolerance to UV and mitomycin C which causes formation of DNA adducts (Kirti et al., 2013).

The *ruvABC* genes are present in all cyanobacteria, to the noticeable exception of *G. kilaueensis* JS1 which lacks *ruvC* (Supplemental Table 1). The *ruvA* and *ruvB* genes are not adjacent unlike their operonic *E. coli* counterparts. Furthermore, *ruvA* is duplicated in *Trichodesmium erythraeum* ISM101, while *ruvC* is quadruplicated in *Cyanothece* ATCC51142 and quadruplicated in *Cyanothece* PCC7822 (Supplemental Table 1). In *Synechocystis* PCC6803 *ruvB* was shown to be dispensable to cell growth in standard laboratory conditions, and to operate in the resistance to UV and H_2_O_2_ (Domain et al., 2004).

Unlike *recAN* and *ruvABC, xerC* is a rare gene in cyanobacteria (Supplemental Table 1). It occurs in a single copy in a few strains, as UCYN-A, *Synechococcus* PCC7942, *Synechococcus* PCC7002 and *Synechocystis* PCC6803, or in several copies in *Cyanothece* PCC7424, *Cyanothece* PCC7822 (two copies), *Nostoc* (*Anabaena*) PCC7120 (three copies), *A. marina* MBIC11017 (eight copies).

In bacteria, homologous recombination preferentially initiates at highly repeated, oligomeric DNA sequences designated as Chi (crossover hotspot instigator) sites. In *E. coli*, the Chi site used by RecBCD is 8 bases (GCTGGTGG), whereas in *B. subtilis* Chi used by AddAB is just 5 bases (AGCGG) (Wigley, 2013). Similarly, the GCGATCGC sequence is overrepresented in many cyanobacteria where one or more methylases recognize some portion of the sequence (Elhai, 2015). In *Synechocystis* PCC6803 the repeated sequence HIP1 (Highly Iterated Palindrome) is associated to a CGATCG-specific methylase (M.Ssp6803I) that is required for rapid growth (Elhai, 2015).

### Distribution of mutagenic DNA repair genes in cyanobacteria

The above-mentioned repair systems usually remove the initial DNA lesions and restore the genetic material back to its original state. When facing many DNA injuries cells start synthesizing several proteins (endonucleases, polymerases and ligases) to accelerate DNA repair, even though there may be some incorporated errors. In this case, the replicative DNA polymerase PolIII, which cannot replicate damaged DNA, is replaced by other polymerases PolIV (encoded by *dinB*) and PolV (encoded by *umuCD*), which replicate damaged DNA in a mutagenic manner (Baharoglu and Mazel, 2014).

The *umuCD* genes (Table 1) are unevenly distributed in cyanobacteria (Supplemental Table 1). Many strains have no *umuCD*, like UCYN-A (small genome) and *Synechococcus* PCC7002. Others possess *umuCD*, such as *Nostoc* (*Anabaena*) PCC7120, *Synechococcus* PCC7942, *Synechocystis* PCC6803, and the *Prochlorococcus* strains. A few strains harbor a duplication of *umuC* (*Synechococcus* PCC6312) and/or *umuD* (*Cyanobium gracile* PCC6307 and *Synechococcus* PCC6312). *A. marina* MBIC11017 possesses three *umuC* and four *umuD* (Swingley et al., 2008). In some cyanobacteria *umuDC* are clustered together, nearby *ruvA* (cluster z in Supplemental Table 1 and Supplemental Figure 1).

The gene *dinB* (Table 1) is present in a very few cyanobacteria, such as *A. marina* MBIC11017, *Anabaena* PCC7120 and *G. kilaueensis* JS1 (Supplemental Table 1).

### Distribution of the key *E. coli*-type SOS genes *LexA* and *SulA* in cyanobacteria

In many bacteria, the so-called “SOS” regulatory system is the main transcriptional circuit that detects DNA damages and regulates the repair systems according to cells needs (Baharoglu and Mazel, 2014). The SOS response is activated when RecA binds single-stranded DNA and generates a nucleofilament triggering the auto-proteolysis of the LexA regulator. In *E. coli*, LexA normally represses about 40 SOS genes (*recABCD, ruvABC*, etc.) by binding to its cognate LexA-box sequence on their promoters (5′-taCTGTatatatatACAGta-3′; the upper cases indicate the conserved nucleotides), thereby precluding their transcription (Baharoglu and Mazel, 2014). One of the SOS-controlled gene codes for the key SulA protein that delays cell division until DNA damages are repaired.

The *lexA* gene (Table 1) is unevenly distributed in cyanobacteria. It is absent in both *Arthrospira* PCC8005 (Badri et al., 2015) and NIES39, and in several strains of the genus *Gloeobacter, Oscillatoria* and *Synechococcus* (including *Synechococcus* PCC7942, Supplemental Table 1), similarly to what found in other bacteria as *H. pylori* (Dorer et al., 2011) and *Streptococcus pneumoniae* (Baharoglu and Mazel, 2014). By contrast, *lexA* is present in the other tested cyanobacteria (it is duplicated in *Cyanothece* ATCC51142). The marine cyanobacteria of the genus *Prochlorococcus* and *Synechococcus* share a very similar *lexA* (clade C), while other strains possess a slightly different *lexA* (clade B), such as *A. marina* MBIC11017, and both *Nostoc* PCC7120 and *Synechocystis* PCC6803 (Li et al., 2010). Interestingly, the *Synechocystis* PCC6803 *lexA* gene appeared to regulate carbon assimilation (Domain et al., 2004) and cell motility (Kizawa et al., 2016), but not DNA recombination and repair (Domain et al., 2004). Furthermore, the *Nostoc* PCC7120 LexA protein has a RecA-independent autoproteolytic cleavage (Kumar et al., 2015).

The *sulA* homolog is present in almost all cyanobacteria, to the noticeable exception of *Gloeobacter violaceus* PCC7421, *G. kilaueensis* JS1, *Anabaena* sp. 90 and UCYN-A (Supplemental Table 1). In *Synechocystis* PCC6803, *sulA* appeared to be indispensable to cell life and division (Raynaud et al., 2004).

### The DNA repair genes present in all cyanobacteria likely encode the core process

Many genes are present in the 76 studied cyanobacteria (*mutM, radA, recA, recFO, recG, recN, ruvABC, ssb*, and *uvrABCD*; Table 1 and Supplemental Table 1), including the marine strain UCYN-A that possesses the smallest genome (1.44 Mb), and numerous marine strains *Prochlorococcus* and *Synechococcus* also endowed with a small genome (1.65–Mb). Similarly, *mutS, recN*, and *ruvC* are present in almost all cyanobacteria (Supplemental Table 1), namely *Thermosynechococcus* NK55a (absence of *mutS1*), *Cyanothece PCC51142* (absence of *recN*) and (*G. kilaueensis* JS1 absence of *ruvC*). Consequently, we propose that the genes *mutMS, radA, recA, recFO, recG, recN, ruvABC, ssb*, and *uvrABCD* encode the core DNA repair system of cyanobacteria.

A few other genes are also very well conserved (Supplemental Table 1), such as *recR* (absent in *C. stagnale* PCC7417, *Cyanobacterium aponinum* PCC10605 and *C. epipsammum* PCC9333), *phr* (absent in *A. marina* MBIC11017, and four *Prochlorococcus* strains: *SS120*, MIT9211, MIT9303 and MIT9313), and *sulA* (absent in UCYN-A, *Anabaena* sp. 90, and the two *Gloeobacter* strains *G. violaceus* PCC7421 and *G. kilaueensis* JS1).

By contrast, mutH is absent in all cyanobacteria (Supplemental Table 1) while *dinB* occurs in only five cyanobacteria (*G. kilaueensis* JS1, *Nostoc* (*Anabaena*) PCC7120, *N. punctiforme* PCC73102, Rivularia PCC7116 and *A. marina* MBIC11017), and *recC* occurs mostly in the marine *Prochlorococcus* and *Synechococcus* strains.

### *Acaryochloris marina* MBIC11017 possesses the largest panel of DNA repair genes some of which occurring in multiple copies in the chromosome and/or plasmids

The cyanobacteria *A. marina* are unique in that they use chlorophyll d to absorb far-red light for photosynthesis. *A. marina* MBIC11017 possesses a large genome (836 Mb) comprising a circular chromosome (6.5 Mb) and nine plasmids [2.13–374 Kb, (Swingley et al., 2008)]. Consistent with its large genome size, *A. marina* MBIC11017 possesses almost all DNA repair genes observed in cyanobacteria, to the noticeable exception of *recC*. In addition to the core genes (*mutMS, radA, recA, recFO, recG, recN, ruvABC, ssb*, and *uvrABCD*) *A. marina* MBIC11017 has the following genes *alkB, dinB* (rare in cyanobacteria), *lexA, mutLTY, phr, ogt, mutLTY, recJQR, sulA, ssb, umuCD*, and *xerC* (Supplemental Table 1). Several of these genes occur in multiple copies (some located on plasmids): *alkB* (two copies), *mutS* (two copies), *ogt* (three copies), *recA* (seven copies, four of them located on four distinct plasmids), *recD* (three copies, two of them propagated on plasmid), *recJ* (two copies), *recQ* (two copies), *ssb* (two copies), *umuC* (three copies including two plasmid copies), *umuD* (four copies including two plasmid copies), and *xerC* (eight copies, including six on plasmids).

The role of the DNA repair genes of *A. marina* MBIC11017 cannot be studied in this host because it has no genetic system yet. However, these genes can be studied in the genetic models *Synechocystis* PCC6803, *Synechococcus* PCC7942, *Synechococcus* PCC7002 or *Nostoc* (*Anabaena*) PCC7120, and their future DNA repair mutants. Hence, it would be interesting to study (and compare) the capability of each of the seven *A. marina* MBIC11017 *recA* genes to complement the detrimental absence of the endogenous *recA* gene of *Synechococcus* PCC7002 (Murphy et al., 1990). If so, the responses of the resulting mutants to DNA damaging agents could be further studied and compared to those of the *Synechococcus* PCC7002 wild-type strain.

### Together, the evolutionary-distant genetic models *Synechocystis* PCC6803, *Synechococcus* PCC7942, *Synechococcus* PCC7002 and *Nostoc* (*Anabaena*) PCC7120 possess almost all DNA repair genes

The cyanobacterial core DNA repair genes (*mutMS, radA, recA, recFO, recG, recN, ruvABC, ssb*, and *uvrABCD*) can be investigated in any genetic models *Synechocystis* PCC6803, *Synechococcus* PCC7942, *Synechococcus* PCC7002 and/or *Nostoc* (*Anabaena*) PCC7120, through deletion and/or over-expression, and phenotypic analysis of the resulting mutants (resistance to DNA damaging agents, etc).

Besides the core DNA repair genes, *Synechocystis* PCC6803, the best-studied model, can be used to investigate *alkB, lexA, mutL, mutS* (a second copy), *mutT, phr, recBcy, recD*, rec*Qcy, sulA, umuC* (two copies), and *umuD* (Supplemental Table 1). The genes missing in *Synechocystis* PCC6803 (*dinB, ogt, mutY, recBec, recC, recJcy, recJec*, and *recQec*) can be studied in the other models (Supplemental Table 1): *Synechococcus* PCC7942 (*mutY, recB*_*ec*_, and the two copies of *ogt* and *recJ*), *Synechococcus* PCC7002 (*mutY, recB*_*ec*_, the two copies of *recJ* and *recQ*_*ec*_) and *Nostoc* PCC7120 (*dinB, ogt*, the two copies of *recJ*, and *recQ*_*ec*_). By contrast, *recC* in occurring only in the marine cyanobacteria *Synechococcus* and *Prochlorococcus*, with no genetics, cannot be studied in its truly natural genetic context. Nevertheless, *recC* can be investigated in any model cyanobacteria mentioned above.

So far only the *ruvB* and *lexA* genes of *Synechocystis* PCC6803 have been studied *in vivo*. While *ruvB* was found to operate in DNA-recombination, *lexA* appeared to regulate carbon assimilation (Domain et al., 2004) and cell motility (Kizawa et al., 2016) but not DNA repair (Domain et al., 2004).

### The *E.coli*-like SOS model for DNA repair is possibly valid for the marine *Prochlorococcus* and *Synechococcus* cyanobacteria, but not for *Gloeobacter, Synechocystis* PCC6803, and *Synechococcus* PCC7942

In addition to the core DNA repair genes (*mutMS, radA, recA, recFO, recG, recN, ruvABC, ssb, and uvrABCD*) the small genomes (1.6–2.7 Mb) of the marine cyanobacteria *Prochlorococcus* and *Synechococcus* possess several genes frequently absent in larger cyanobacterial genomes (*recBCD* and *umuCD*; Supplemental Table 1). *Prochlorococcus* and *Synechococcus* also have homologs of *lexA* and *sulA*, which encode the key *E. coli* SOS proteins LexA (regulation of the SOS system) and SulA (postponing of cell division until completion of DNA reparation) (Baharoglu and Mazel, 2014). Furthermore, *recA* and *uvrA* are induced by UV in *Prochlorococcus* and *Synechococcus* (no information is provided for the other genes), as occurs in *E. coli* (Mella-Flores et al., 2012). The distribution of DNA repair genes in *Prochlorococcus* and *Synechococcus* marine strains suggest that they may possess an *E.coli*-like SOS system. This hypothesis is consistent with the fact that the mutation rate of *Prochlorococcus* is similar to that of *E. coli* (Biller et al., 2015).

By contrast, several findings indicate that the *E.coli*-like SOS model for DNA repair is not valid for all cyanobacteria. The strongest evidence is that two cyanobacteria *G. violaceus* PCC7421 and *G. kilaueensis* JS1 have none of the two key SOS genes *lexA* and *sulA*, and they also lack *alkB, recBC* and *xerC* (Supplemental Table 1). Similarly, *Synechococcus* PCC7942 (and its sister strain PCC6301) has no *lexA, alkB, dinB*, and *recCD*, while *Anabaena* sp. 90 lacks *sulA, dinB, ogt, recBCD* and *umuCD*. *Synechocystis* PCC6803 possesses *lexA*, but it does not regulate DNA repair genes; it controls carbon assimilation (Domain et al., 2004) and cell motility (Kizawa et al., 2016). Furthermore, the *Synechocystis* PCC6803 *lexA* and *recA* genes are not induced by UV-C as occur in *E. coli*, actually they are downregulated by UV-C (Domain et al., 2004) [*lexA* is also negatively regulated by UV-B (Huang et al., 2002)]. In addition, the *Synechocystis* PCC6803 *lexA* and *recA* promoters have neither *E. coli*-like nor *B. subtilis*-like SOS boxes (Domain et al., 2004). Similarly, no SOS box was found in the promoter region of the *Synechococcus* PCC7002 *recA* gene (Murphy et al., 1990). Furthermore, the *lexA* gene of *Anabaena* PCC7120 was neither induced by UV-B nor mitomycin C. In addition, the *Synechocystis* PCC6803 LexA protein has a RecA-independent autoproteolytic cleavage (Kumar et al., 2015).

In *Synechococcus* PCC7942, the Weigle-reactivation of irradiated phage (As-1) was neither induced by mitomycin-C nor nalidixic acid, unlike what was found in *E.coli* (Lanham and Houghton, 1988).

## Conclusion

From bacteria to higher eukaryotes, cells are equipped with various conserved systems to repair DNA damages generated by their own metabolism (Imlay, 2013) or exogenous sources (solar UV, gamma radiations, chemicals, etc.). Inevitably, some DNA lesions are not correctly repaired leading to mutations that can influence cell fitness (Baharoglu and Mazel, 2014).

For historical reasons, DNA recombination and repair in prokaryotes have been mostly studied in the (non-photosynthetic) bacterium *E. coli* (Baharoglu and Mazel, 2014). Unlike *E.coli*, cyanobacteria are continuously exposed to DNA damages generated by solar UV rays and their own photosynthetic metabolism (Cassier-Chauvat and Chauvat, 2015). As a likely consequence, all tested cyanobacteria were found to be more radiation resistant than *E. coli*. It is also important to study DNA recombination and repair in cyanobacteria for biotechnological purposes, since many recombinant strains appeared to be genetically unstable. They somehow managed to inactivate the (newly-introduced) heterologous genes of industrial interest. Thus, a better understanding of DNA recombination and repair in cyanobacteria may lead to increasing the genetic stability of biotechnologically important strains, an important industrial goal.

Using a comparative genomic approach, we found that cyanobacteria possess many genes orthologous to *E. coli* DNA recombination and repair genes, notwithstanding the possibility that cyanobacteria have other, as yet unidentified, such genes.

These *E. coli*-like genes are unevenly distributed in cyanobacteria, in agreement with their wide genome diversity, in a way consistent with the size of their genomes, i.e., large genomes tend to possess more DNA repair genes than small genomes. Most of these *E. coli*-like genes are scattered throughout cyanobacterial genomes, suggesting that there is a mechanism for their coordinate regulation or that they are mostly expressed constitutively. Many DNA repair genes (*mutMS, radA, recA, recFO, recG, recN, ruvABC, ssb*, and *uvrABCD*) are extremely well conserved in cyanobacteria, including in the *Prochlorococcus* and *Synechococcus* marine strains which possess very small genomes (1.44–2.7 Mb). Consequently, we propose that these genes encode the core DNA repair system of cyanobacteria.

These marine *Prochlorococcus* and *Synechococcus* cyanobacteria also have the genes *recBCD* (DNA recombination), *umuCD* (mutational DNA replication), and the key SOS genes *lexA* (regulation of the SOS system) and *sulA* (postponing of cell division until completion of DNA reparation). These findings suggest that the marine *Prochlorococcus* and *Synechococcus* cyanobacteria may possess an *E. coli*-type SOS system.

In contrast, other cyanobacteria endowed with larger genomes lack some of the SOS key genes (*lexA, sulA, recBCD*, or *umuCD*). For instance, *G. violaceus* PCC7421 and *G. kilaueensis JS1* lack *lexA, recBC*, and *sulA* (they also lack *alkB* and *xerC*). *Synechococcus* PCC7942 has neither *lexA* nor *recCD*. Furthermore, the *lexA* gene of *Synechocystis* PCC6803 is not involved in the regulation of DNA repair genes (Domain et al., 2004). Collectively, these findings suggest that the *E.coli*-like SOS model for DNA repair is likely not valid for all cyanobacteria.

The cyanobacterium *A. marina* MBIC11017 possesses the most complete, and complex, set of DNA repair genes: *alkB* (two copies), *dinB* (rare in cyanobacteria), *lexA, mutL, mutM, mutS* (two copies), *mutT, mutY, ogt* (three copies), *phr, radA, recA* (seven copies, four of them located on plasmids), *recD* (three copies, including two plasmidic copies), *recF, recG, recJ* (two copies), *recN, recO, recQ* (two copies), *recR, ruvABC, ssb* (two copies), *sulA, umuC* (three copies including two plasmid copies), *umuD* (four copies including two plasmid copies), *uvrABCD* and *xerC* (eight copies, including six on plasmids). However, *A. marina* MBIC11017 has not all DNA repair genes, since it lacks *recC*. All cyanovacterial DNA repair genes naturally present (or not) in the few (evolutionary distant) genetic models *Synechocystis* PCC6803, *Synechococcus* PCC7002, *Synechococcus* PCC7942 and *Nostoc* (*Anabaena*) PCC7120, can be studied through deletion and/or over-expression, and analysis of the corresponding mutants (e.g., resistance to DNA damaging agents). Such works would be most welcome since little is known about DNA recombination and repair in cyanobacteria. So far, only the *recA, ruvB*, and *lexA* genes have been studied *in vivo*. The *recA* gene appeared to be indispensable in *Synechococcus* PCC7002 (Murphy et al., 1990), and dispensable in *Synechocystis* PCC6803 (Minda et al., 2005). The *Synechocystis* PCC6803 *recA*-null mutant was sensitive to UV-C and white light. The *Synechocystis* PCC6803 *ruvB* gene was found to operate in DNA-recombination, while *lexA* appeared to regulate carbon assimilation (Domain et al., 2004) and cell motility (Kizawa et al., 2016), but not DNA repair (Domain et al., 2004). We hope that this review will stimulate future studies of DNA recombination and repair in cyanobacteria so as to answer the following questions, among others. Do cyanobacteria possess DNA recombination and repair genes with no counterpart in a non-photosynthetic and radiation-sensitive bacterium such as *E. coli*? What is the specificity/redundancy of the various copies of the repeated genes of cyanobacteria (for example of the seven *recA* genes of *A. marina* MBIC11017)? What are the molecular mechanisms responsible for the high radiation-resistance of some cyanobacteria (for instance *Chroococcidiopsis*). How to improve the genetic stability of cyanobacterial strains engineered for biotechnological puproses?

## Author contribution

CC and FC conceived the study. CC, TV, and FC carried out the literature search and analyzed the data. CC, TV, and FC wrote the paper.

### Conflict of interest statement

The authors declare that the research was conducted in the absence of any commercial or financial relationships that could be construed as a potential conflict of interest.

## Supplementary material

Supplementary Table 1 **Distribution of DNA repair genes in cyanobacteria**. The presence (indicated by the number of copies) or absence (0) of the gene is indicated along with the letters referring to the conserved gene clusters depicted in Supplemental Figure 1.

Supplementary Figure 1 **Conserved genomic organization around the DNA repair genes in cyanobacterial genomes**. Genes are represented by boxes pointing in the direction of their transcription. DNA repair genes are colored in red. Genes encoding hypothetical proteins are indicated as “ho.”
