# Supplementary material for: Comparative Genomics of DNA Recombination and Repair in Cyanobacteria: Biotechnological Implications
Source: Front Microbiol. 2016 Nov 9;7:1809. doi: 10.3389/fmicb.2016.01809 (PMC5101192; doi:10.3389/fmicb.2016.01809)
Supplement: Supplementary Figure 1 — Conserved genomic organization around the DNA repair genes in cyanobacterial genomes. Genes are represented by boxes pointing in the direction of their transcription. DNA repair genes are colored in red. Genes encoding hypothetical proteins are indicated as “ho.” [file Image1.pdf]

uvr genes

Cluster a

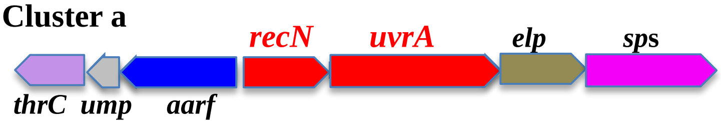

Cluster b

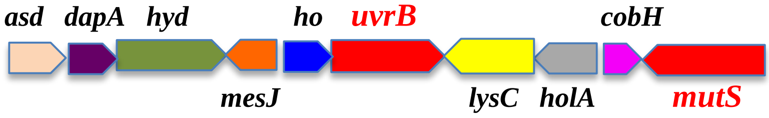

Cluster c

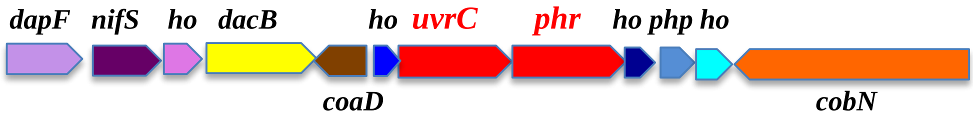

Cluster d

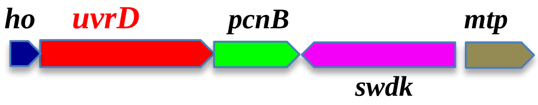

rec genes

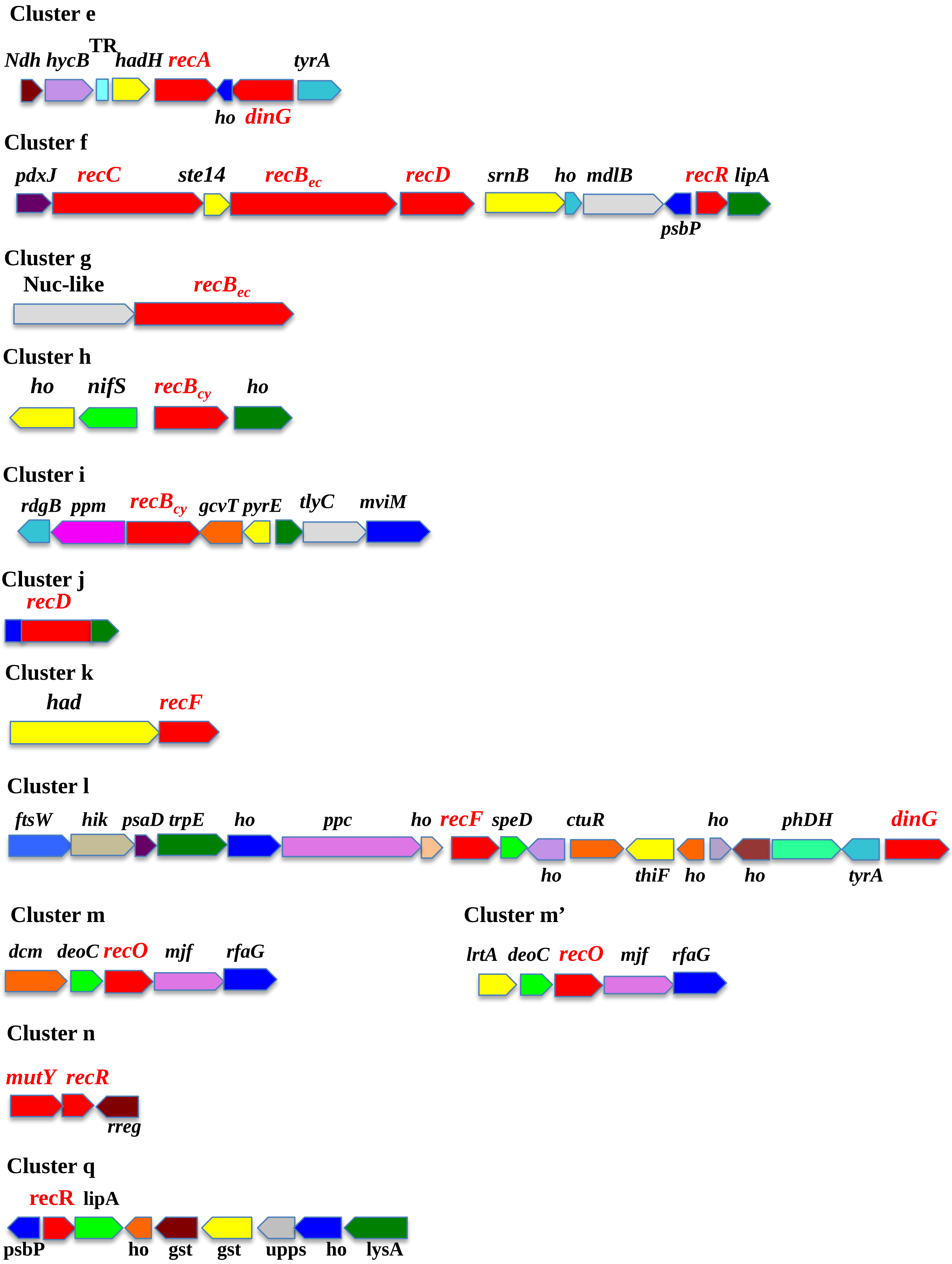

Cluster e
Ndh hycB TR hadH recA tyrA
ho dinG
Cluster f
pdxJ recC ste14 $recB_{ec}$ recD srnB ho mdlB recR lipA
psbP
Cluster g
Nuc-like $recB_{ec}$
Cluster h
ho nifS $recB_{cy}$ ho
Cluster i
rdgB ppm $recB_{cy}$ gcvT pyrE tlyC mviM
Cluster j
recD
Cluster k
had recF
Cluster l
ftsW hik psaD trpE ho ppc ho recF speD ctuR ho phDH dinG
ho thiF ho ho tyrA
Cluster m
dcm deoC recO mjf rfaG
Cluster m'
lrtA deoC recO mjf rfaG
Cluster n
mutY recR
rreg
Cluster q
recR lipA
psbP ho gst gst upps ho lysA

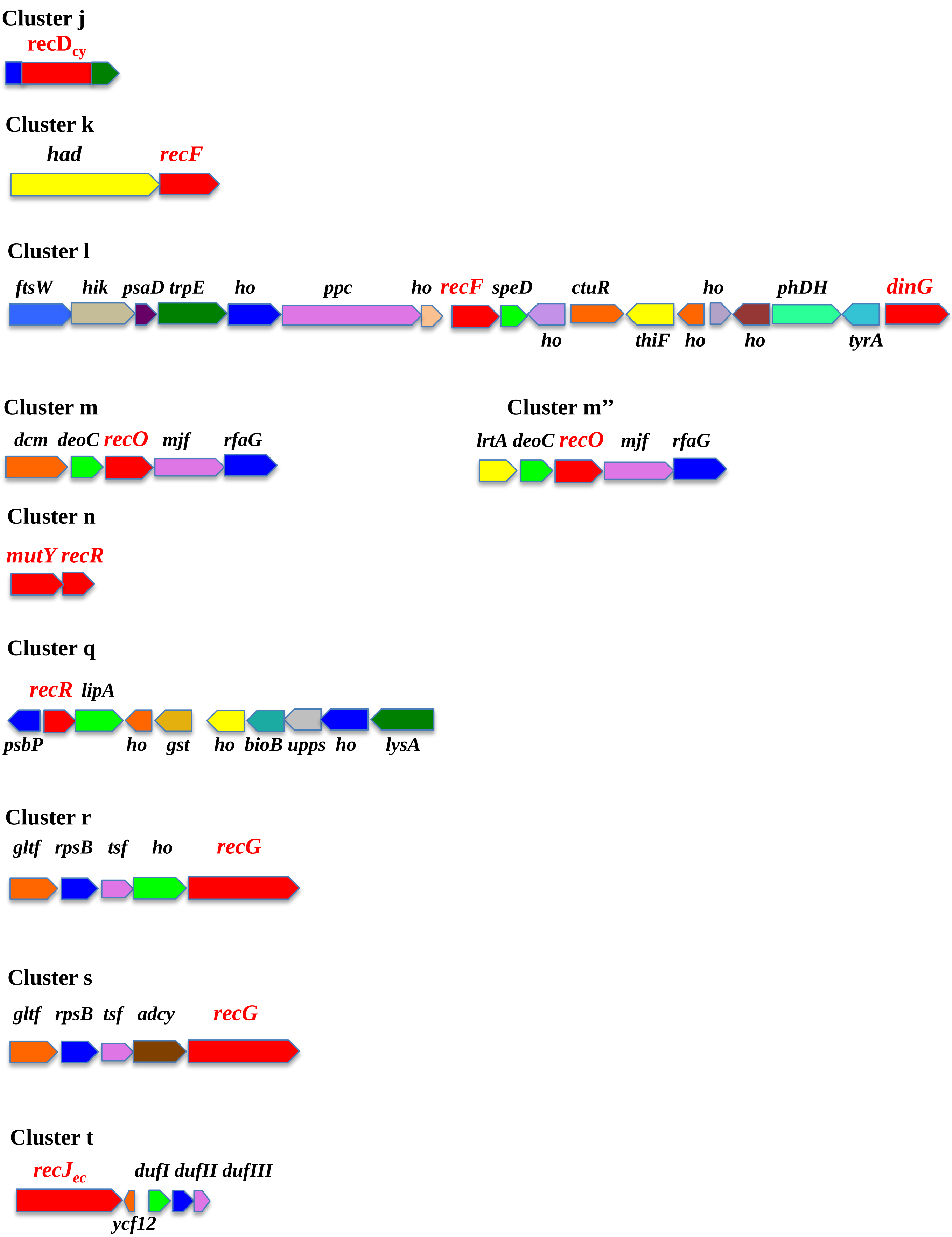

Cluster j
recDcy
Cluster k
had
recF
Cluster l
ftsW
hik
psaD
trpE
ho
ppc
ho
recF
speD
ctuR
ho
phDH
dinG
ho
thiF
ho
ho
tyrA
Cluster m
dcm
deoC
recO
mjf
rfaG
Cluster m''
lrtA
deoC
recO
mjf
rfaG
Cluster n
mutY
recR
Cluster q
recR
lipA
psbP
ho
gst
ho
bioB
upps
ho
lysA
Cluster r
gltf
rpsB
tsf
ho
recG
Cluster s
gltf
rpsB
tsf
adcy
recG
Cluster t
recJec
dufI
dufII
dufIII
ycf12

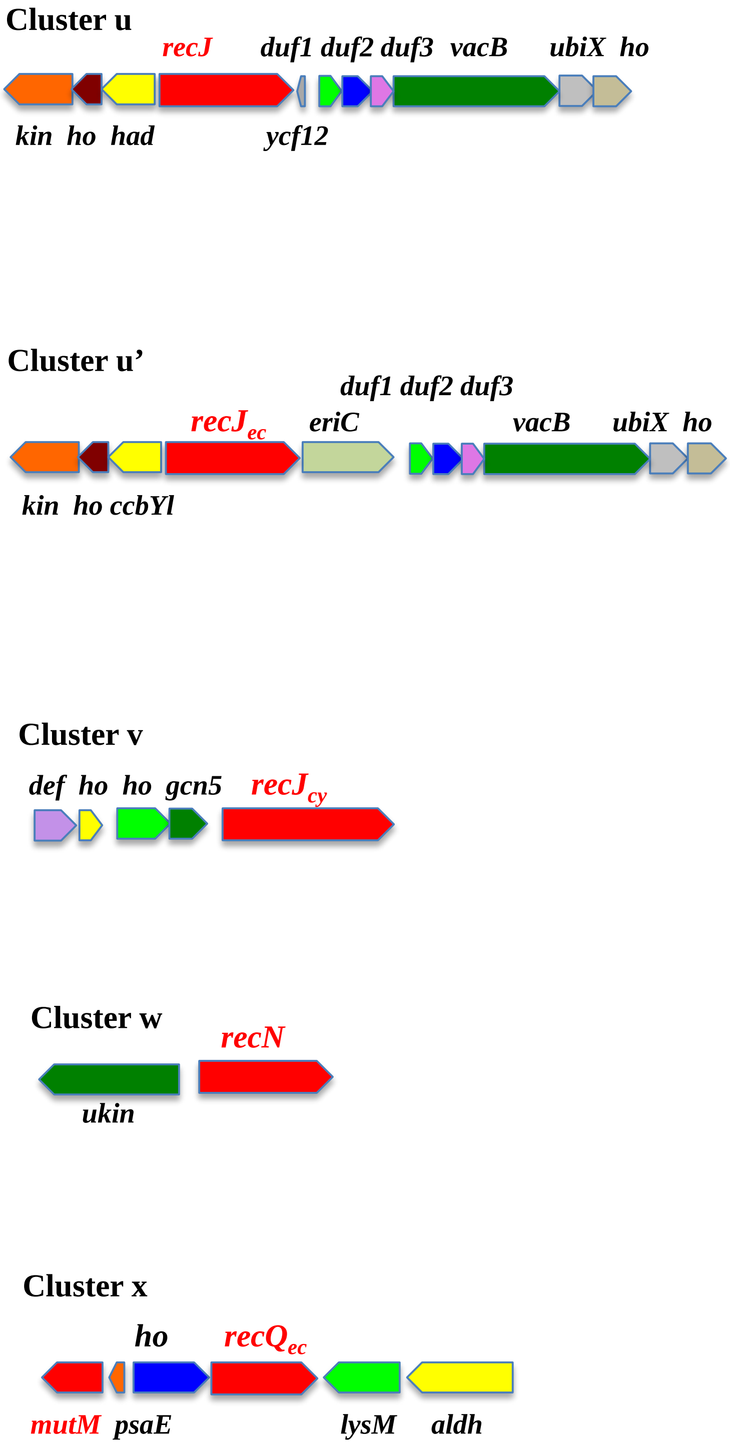

Cluster u
recJ
duf1 duf2 duf3 vacB
ubiX ho
kin ho had
ycf12
Cluster u'
duf1 duf2 duf3
recJ$_{ec}$
eriC
vacB
ubiX ho
kin ho ccbYl
Cluster v
def ho ho gcn5
recJ$_{cy}$
Cluster w
recN
ukin
Cluster x
ho
recQ$_{ec}$
mutM psaE
lysM aldh

Cluster y

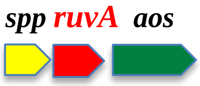

Cluster z

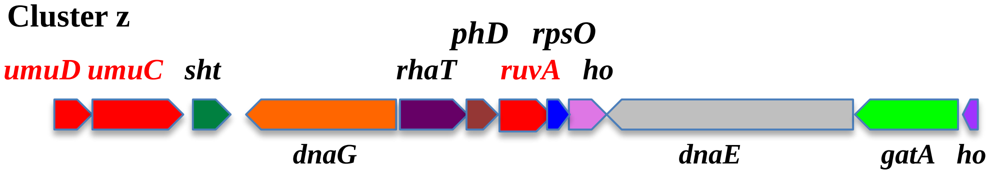

Cluster ab

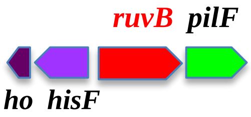

Cluster ac

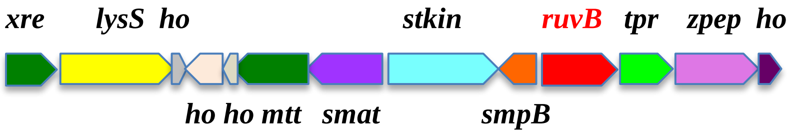

Cluster ad

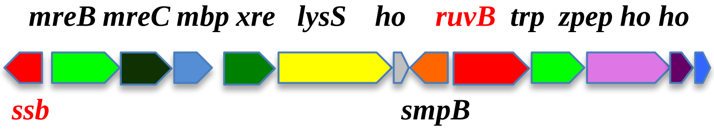

Cluster ae

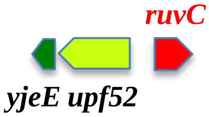

Cluster af

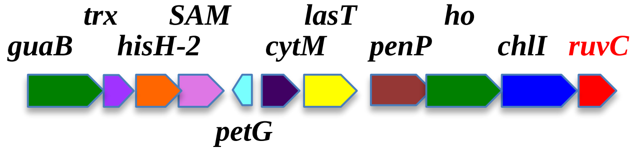

Cluster af[$]

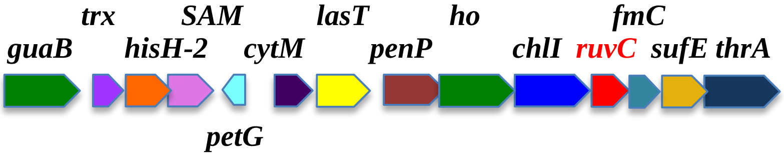

Cluster ag

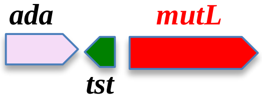

Cluster ah

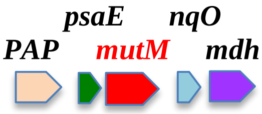

Cluster ai et ai’

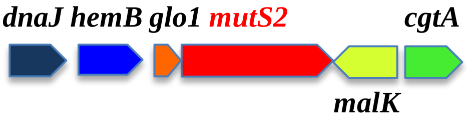

Cluster aj

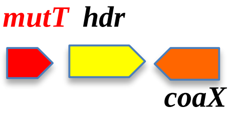

Cluster ak

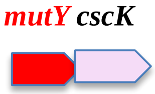

Cluster al

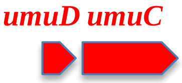

Cluster am

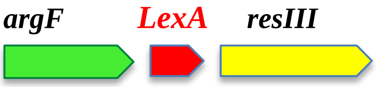

Cluster an

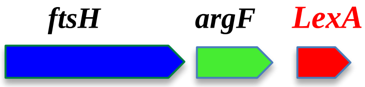

Cluster ao

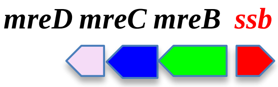

Com genes

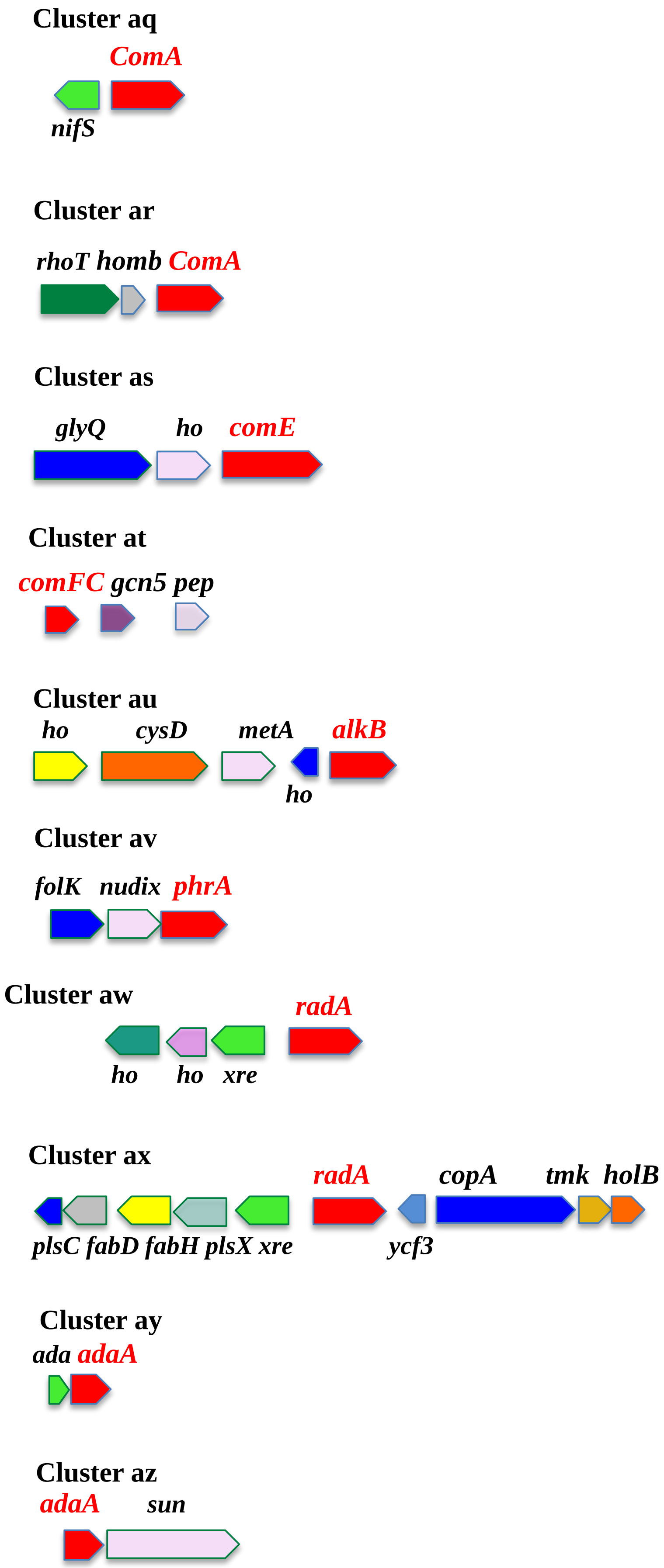
Cluster aq
ComA
nifS
Cluster ar
rhoT homb ComA
Cluster as
glyQ ho comE
Cluster at
comFC gcn5 pep
Cluster au
ho cysD metA alkB
ho
Cluster av
folK nudix phrA
Cluster aw
radA
ho ho xre
Cluster ax
radA copA tmk holB
plsC fabD fabH plsX xre ycf3
Cluster ay
ada adaA
Cluster az
adaA sun

- **Supplemental Fig.1: Conserved genomic organization around the DNA repair genes in cyanobacterial genomes.**
- Genes are represented by boxes pointing in the direction of their transcription. DNA repair genes are colored in red. Genes encoding hypothetical proteins are indicated as “ho”.
- **Cluster a:** thrC: threonine synthase; ump: uncharacterized membrane protein; aarF: protein kinase; recN; uvrB; elp: esterase-lipase protein; sps: sucrose phosphate synthase. **Cluster b:** asd: aspartate semialdehyde dehydrogenase; dapA: dihydrodipicolinate synthase; hyd: metallo-beta-lactamase hydrolase; mesJ: ATPase; uvrB; lysC: aspartate kinase; holA: DNA polymerase III subunit; CobH: precorrin-8X methylmutase; mutS. **Cluster c:** dapF: diaminopimelate epimerase; nifS: cysteine desulfurase; ho; dacB: D-alanyl-D-alanine carboxypeptidase; coaD phosphopantetheine adenylyltransferase; uvrC; phr; PHP metal-dependent phosphoesterase; cobN: cobalamin biosynthesis protein. **Cluster c’**: cluster c without phr. **Cluster d:** uvrD; pcnB: tRNA nucleotidyltransferase/poly(A) polymerase; swdk: selenide water dikinase; mtp : methylthioadenosine phosphorylase. **Cluster e:** Ndh: NAD(P)H-quinone oxidoreductase; hycB: Fe-S-hydrogenase; TR tRNA-Gln; hadh: haloacid dehalogenase; recA; dinG: DNA helicase; tyrA: arogenate dehydrogenase.
- **Cluster f:** pdxJ: pyridoxine 5'-phosphate synthase; recC; ste14: S-isoprenylcysteine methyltransferase; recBec; recD; srnB: RNA helicase; mdlB: ABC-type transport; psbP: photosystem II protein; recR; lipA: lipoate synthase. **Cluster g**: nuc-like: ATP dependant nuclease; recBec. **Cluster h:** nifS: L-cysteine/cystine lyase; $recB_{cy}$. **Cluster i:** rgd: deoxyribonucleotide pyrophosphatase; manB: phospho-mannomutase; gcvT: aminomethyl transferase; pyrE: orotate phosphoribosyl-transferase; tlyC: hemolysin; mviN: oxidoreductase. **Cluster j:** recD-like protein. $: recD behind recA. **Cluster £**: *recF* upstream of *radA*. **Cluster k:** had: haloacid dehalogenase; recF. **Cluster l:** ftsW: cell division protein; hik: histidine kinase; psaD: photosystem I protein; trpE: anthranilate synthase; ppc: phosphoenol-pyruvate carboxylase; recF; speD: S-adenosylmethionine decarboxylase; ctuR: corrinoid adenosyltransferase; thiF: molybdopterin biosynthesis; phdh: phytoene dehydrogenase; tyrA : arogenate dehydrogenase; dinG : Rad3-related helicase. **Cluster m:** dcm: cytosine methyltransferase; deoC: deoxyribose-phosphate aldolase; recO; mjf: facilitator transporter; rfaG: glycosyltransferase. **Cluster m’:** cluster m without dcm. **Cluster m’*:** cluster m’ without rfaG. **Cluster m”:** cluster m where dcm replaces lrtA: light-repressed protein. **Cluster m*:** cluster m without dcm and deoC. **Cluster m**:** cluster m* without rfaG. **Cluster m***:** only deoC upstream recO. **Cluster m’”:** Cluster m’’ without mjf. **Cluster n:** mutY; recR. **Cluster q:** shares genes with cluster f. psbP; recR; lipA: lipoate synthase; gst: glutathione S-transferase; bioB: biotin synthase; upps: UDP pyrophosphate synthase; lysA: diaminopimelate decarboxylase. **Cluster q’**: cluster q without gst. **Cluster q”**: cluster q without ho between psbP and recR. **Cluster r:** gltf: glycosyl transferase; rspB: 30S ribosomal protein; tsf: elongation factor. **Cluster r*:** cluster r with insertion between gltf and rpsB. **Cluster r’:** cluster r without gltf. **Cluster r’’:** cluster r without gltf, rpsB, tsf. **Cluster s:** cluster r where recG is replaced by adcy: adenylate cyclase. **Cluster t**: $recJ_{ec}$; ycf12: photosystem II protein; duf1: UPF0153 Fe-S protein; duf2: UPF0016 protein; duf3: conserved domain protein. **Cluster t’:** cluster t with insertion between duf1 and duf2. **Cluster t*:** cluster t without ycf12. **Cluster u:** cluster t harboring kin (kinase) and had (putative haloacid dehalogenase) upstream of recJ and ubiX and ho downstream duf3; UbiX: amino acid decarboxylase. had; vacB: acetazolamide resistance Zam/organic solvent ABC permease. **Cluster u’**: cluster u with eriC (chloride channel) replacing ycf12. **Cluster u”**: cluster u without kin.

- **Cluster v**: def: peptide deformylase; gcn5: N-acetyltransferase. **Cluster v***: cluster v with an insertion between the two ho. **Cluster v'**: cluster v without def and ho. **Cluster v"**: cluster v without gcn5. **Cluster w:** ukin: unusual protein kinase &: cluster w with mutL downstream recN. **Cluster a:** see uvrA. **Cluster x:** psaE: photosystem I centre. mut M; recQ; LysM; peptidoglycan-binding; aldh; aldehyde dehydrogenase. **Cluster y**: spp: sucrose-phosphate phosphatase; ruvA; aos: 8-amino-7-oxononanoate synthase. **Cluster y':** cluster y without aos. **Cluster y":** cluster y without spp. **Cluster z:** umuD; umuC; sht: serine hydroxymethyltransferase; dnaG: DNA primase; rahT: putative transporter; phD: cAMP phosphodiesterase; ruvA; rpso: ribosomal protein S15; dnaE: DNA polymerase III; gatA: glutamyl-tRNA(Gln) amidotransferase. ho. **Cluster z**': cluster z without umuCD and sht. **Cluster z**": cluster z with only rpso and ho downstream ruvA. **Cluster z*:** cluster z with various genes upstream of dnaG and RuvA. **Cluster ab:** hisF: imidazole glycerol phosphate synthase; ruvB; pilF: pilus assembly. **Cluster ab':** cluster ab without pilF. **Cluster ab":** cluster ab without hisF. **Cluster ac:** xre: transcriptional regulator; lysS; lysine-tRNA ligase; 3 ho; mtt: methyltransferase; Smat: sulfatase maturase ; stkin : serine/threonine kinase ; smpB: SsrA-binding; ruvB; tpr: TPR repeat-containing protein; zpep: zinc metallopeptidase; ho. **Cluster ad:** cluster ac where genes are replaced by ad: ssb; mreB: rod shape; mreC: rod shape MreB; mbp: membrane protein. **Cluster ae:** yjeE: UPF0079 protein; upf52: UPF0052 protein; ruvC. **Cluster af**: guaB: inosine-5-monophosphate dehydrogenase; trx: thioredoxin; hisH-2: glutamine amidotransferase; SAM: methyltransferase; petG: cytochrome b6-f; cytM: cytochrome C; lasT: rRNA methyltransferase; penP: serine hydrolase; Ho; clhI: Mg-protoporphyrin chelatase; ruvC. **Cluster af'**: cluster af without petG. **Cluster af$^{\$}$**: cluster af with genes downstream of ruvC. fmC; 5-formyltetrahydrofolate ligase; sufE: cysteine desufurase and thrA: homoserine dehydrogenase. **Cluster ag:** ada: adenosine deaminase; tst: rhodanese protein; mutL. **Cluster ag':** cluster ag without ada. **Cluster ag*:** cluster ag genes inserted between ada and tst. **Cluster ah:** PAP: PAP fibrillin; psaE: photosystem I; *mutM*; nqO: NAD(P)H-quinone oxidoreductase; mdh: malte dehydrogenase. **Cluster ai:** dnaJ: chaperone; hemN: delta-aminolevulinic dehydratase; glo: lactoylglutathione lyase ; mutS; malK: sugar transporter. cgtA: GTPase. **Cluster ai':** cluster ai without dnaJ. **Cluster ai":** cluster ai without glo1. **Cluster ai*:** cluster ai without malK. **Cluster ai'*:** cluster ai without both dnaJ and malK. **Cluster aj:** mutT; hdr: alpha/beta hydrolase; coaX: pantothenate kinase. **Cluster aj':** cluster aj without coaX. **Cluster aj':** cluster aj without hdr. **Cluster ak:** mutY; cscK: sugar kinase. **Cluster al:** umuC; umuD. **Cluster am:** argF: ornithine carbamoyltransferase; lexA; resIII: DNA phosphorothioation. **Cluster an:** ftsH: cell division; argF: ornithine carbamoyltransferase; lexA. **Cluster an*:** similar to cluster an with genes inserted between argF and lexA. **Cluster ao:** mreD; mreC; mreB; ssb. **Cluster ao'** cluster ao without mreD mreC. **Cluster aq**: nifS; comA. **Cluster aq'**: cluster aq with ho between nifS and comA. **Cluster ar**: rhoT: rho termination protein; homb hypothetical protein; comA. **Cluster ar***: comA triplicated. **Cluster as**: glyQ: glycyl-tRNA synthetase; ho; comE. **Cluster as'**: Cluster as without ho. **Cluster as"**: Cluster as without glyQ. **Cluster at**: gcn5: GCN5-N-acetyltransferase peptidase; comFC: pep: peptidase. **Cluster au**: cysD: O-acetylhomoserine aminocarboxypropyltransferase; metA : homoserine O-succinyltransferase; ho; alkB: oxidative demethylase. **Cluster av**: folK: 2-amino-4-hydroxy-6- hydroxymethyldihydropteridine pyrophosphokinase; nudix: NTP pyrophosphohydrolase; phrA. **Cluster av'**: Cluster av harboring ho between nudix and phrA. **Cluster av"**: Cluster av without folK. **Cluster aw**: ho; ho; xre: transcriptional regulator; radA. **Cluster aw'**: cluster aw with one ho gene.

- **Cluster aw”**: cluster aw with no ho. **Cluster ax**: plsC: glycerol 3-phosphate transferase; fabD: malonyl CoA transacylase; fabH:3-oxoacyl-ACP synthase; plsX: glycerol-3-phosphate acyl-transferase; xre: transcriptional regulator; radA; ycf3: photosystem I; copA: heavy metal transport; tmk : thymidylate kinase; holB : DNA polymerase III. **Cluster ay:** ada: transcriptional activator: methylated-DNA-cysteine S-methyltransferase. In some cases Ada is fused to AdaA. **Cluster az:** adaA; sun: 16S rRNA methyltransferase.
